# Human but Not Mouse Adipogenesis Is Critically Dependent on LMO3

**DOI:** 10.1016/j.cmet.2013.05.020

**Published:** 2013-07-02

**Authors:** Josefine Lindroos, Julia Husa, Gerfried Mitterer, Arvand Haschemi, Sabine Rauscher, Robert Haas, Marion Gröger, Robert Loewe, Norbert Kohrgruber, Klaus F. Schrögendorfer, Gerhard Prager, Harald Beck, J. Andrew Pospisilik, Maximilian Zeyda, Thomas M. Stulnig, Wolfgang Patsch, Oswald Wagner, Harald Esterbauer, Martin Bilban

**Affiliations:** 1 Department of Laboratory Medicine, Medical University of Vienna, 1090 Vienna, Austria; 2 Department of Dermatology, Medical University of Vienna, 1090 Vienna, Austria; 3 Department of Surgery, Division of Plastic and Reconstructive Surgery, Medical University of Vienna, 1090 Vienna, Austria; 4 Christian Doppler Laboratory for Cardio-Metabolic Immunotherapy at the Department of Internal Medicine III, Medical University of Vienna, 1090 Vienna, Austria; 5 Max Planck Institute of Immunobiology and Epigenetics, 79108 Freiburg, Germany; 6 Institute of Pharmacology and Toxicology, Paracelsus Medical University, 5020 Salzburg, Austria

## Abstract

Increased visceral fat is associated with a high risk of diabetes and metabolic syndrome and is in part caused by excessive glucocorticoids (GCs). However, the molecular mechanisms remain undefined. We now identify the GC-dependent gene *LIM domain only 3* (*LMO3*) as being selectively upregulated in a depot-specific manner in human obese visceral adipose tissue, localizing primarily in the adipocyte fraction. Visceral *LMO3* levels were tightly correlated with expression of 11β-hydroxysteroid dehydrogenase type-1 (*HSD11B1*), the enzyme responsible for local activation of GCs. In early human adipose stromal cell differentiation, GCs induced LMO3 via the GC receptor and a positive feedback mechanism involving 11βHSD1. No such induction was observed in murine adipogenesis. LMO3 overexpression promoted, while silencing of LMO3 suppressed, adipogenesis via regulation of the proadipogenic PPARγ axis. These results establish LMO3 as a regulator of human adipogenesis and could contribute a mechanism resulting in visceral-fat accumulation in obesity due to excess glucocorticoids.

## Highlights

• LMO3 is a target and amplifier of glucocorticoid action in human fat cells
• LMO3 levels are selectively elevated in visceral adipose tissue of obese humans
• LMO3 is required for human but not mouse adipocyte differentiation
• LMO3 promotes adipogenesis by boosting a proadipogenic PPARγ program

## Introduction

The study of human white fat-cell development and distribution has become an important issue in the past decades due to the immense prevalence of obesity and related disease. High risk of developing metabolic disease is associated with amplified visceral (VI) obesity, whereas obese subcutaneous (SC) adipose tissue (SAT) presents a smaller or no risk and may even be protective (Gabriely et al., 2002; Jensen, 2008; Tran et al., 2008). These differences in contribution to disease and function could be caused by regional variations of replication and adipogenic potential. Depot-specific fat mass expansion mechanisms may facilitate drug development targeting particular fat depots. Fat-depot differences in human adipogenic potential (SC > VI [Tchkonia et al., 2005; Tchkonia et al., 2013; Tchoukalova et al., 2010]) have been attributed to depot-specific intrinsic gene expression signatures (Gesta et al., 2006; Macotela et al., 2012; Perrini et al., 2013; Tchkonia et al., 2007) such as higher levels of PPARγ and C/EBPα in differentiating SC human adipose stromal cells (hASCs) and their superior response to troglitazones (TZDs) (Hauner et al., 1988; Tchkonia et al., 2002). Other modulators could be extrinsic factors such as glucocorticoids (GCs), which are known to potentiate human adipogenesis (Morton, 2010; Tomlinson et al., 2006; Wiper-Bergeron et al., 2007). Clinically, GCs are widely used as immunosuppressants directly regulating transcription via the glucocorticoid receptor (GR). The enzyme 11-β-hydroxysteroid dehydrogenase type 1 (11βHSD1) plays a crucial role in determining intracellular (prereceptor level) GC levels by regenerating active GCs (cortisol) from inactive metabolites (cortisone) and is highly expressed in visceral adipose tissue (VAT) (Bujalska et al., 2008; Morton, 2010; Walker and Andrew, 2006). In humans, the adipogenic-enhancing properties of GCs are most obvious in the truncal obesity of Cushing’s syndrome (Arnaldi et al., 2010), as well as in patients on systemic immunosuppressive corticosteroid treatment (McDonough et al., 2008). In common for these individuals are hypertension, VAT expansion, and insulin resistance (Björntorp and Rosmond, 2000).

In this study, we investigated the role of GCs in human adipocyte differentiation by analyzing the transcriptome of GC-induced primary hASCs. This approach identified LMO3, a member of the LIM-only proteins (LMOs), known to be involved in cell-fate determination and neurogenesis (Dawid et al., 1998; Zheng and Zhao, 2007), as a critical GC-responsive proadipogenic regulator. Further, *LMO3* was among the earliest factors induced in the course of human but not mouse white adipocyte differentiation. We demonstrate that LMO3 exerts its activity at the interface between GC action and peroxisome proliferator-activated receptor γ (PPARγ). Importantly, *LMO3* was upregulated in VAT (as compared to SAT) in obese humans and tightly correlated with 11β-hydroxysteroid dehydrogenase type-1 (*HSD11B1*) expression. These findings present LMO3 as a proadipogenic factor that is (1) driven by GC action, (2) showing high expression levels in human VAT, and (3) critical for human adipocyte differentiation. Thus, LMO3 could provide an attractive target to interfere with human adipocyte differentiation in a therapeutically relevant manner.

## Results

### LMO3 Is A Target and Amplifier of GC Action in Human VAT

**Figure 1:**
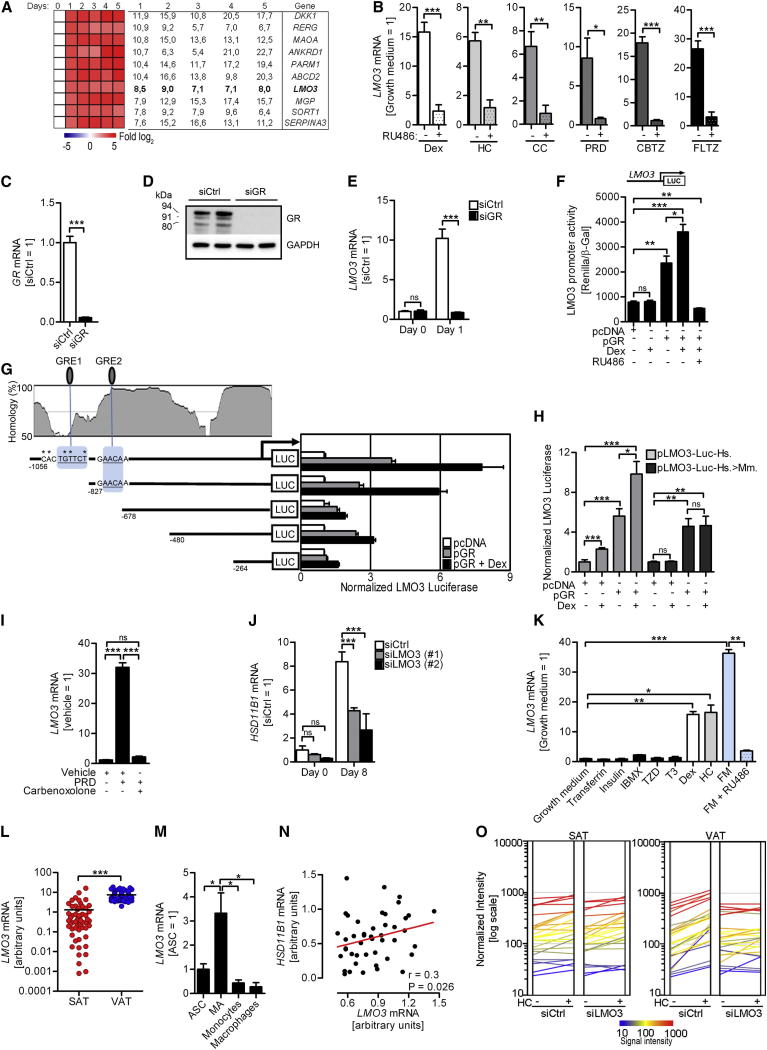
LMO3 Expression Is Regulated by the GC Receptor (A) hASCs were treated with Dex and mRNA isolated on indicated time points. Fold change is compared to day 0 of Dex treatment. (B) *LMO3* expression 24 hr after addition of GCs to hASCs in growth medium ±RU486. *LMO3* in the absence of RU486 in growth medium was set to 1 (n = 3). (C and D) mRNA (C) and protein (D) of GR in transfected hASCs treated for 24 hr with Dex. (E) *LMO3* mRNA in transfected hASCs with Dex treatment for 0 and 24 hr. (F–H) LMO3 promoter analysis. 293FT cells were cotransfected with pcDNA and/or GC receptor expression plasmid (pGR), as well as human full-length LMO3 promoter luciferase reporter plasmid (“LUC”; promoter construct shown above) (F), various deletion constructs (G, right), or LMO3 construct featuring the murine GRE1 site (pLMO3-Luc-Hs.>Mm.) (H). Twenty-four hours after transfection, cells were treated with DMSO, Dex, or RU486. Underlined letters, GRE half sites; ^∗^, point mutations representing the murine sequence. Deletion constructs are shown below homology plot (G, left). (I) *LMO3* mRNA expression in hASCs treated for 24 hr with vehicle, carbenoxolone, or PRD in growth medium. (J) *HSD11B1* mRNA during the indicated days of differentiation in transfected hASCs. (K) *LMO3* mRNA in hASCs after adding individual components of the adipogenic cocktail or full mix (FM) in growth medium for 24 hr. (n = 3). (L) Depot-specific expression of *LMO3* mRNA in paired obese human SAT and VAT biopsies (n = 55). Horizontal bars indicate the mean. (M) Human *LMO3* mRNA expression in human VAT fractions normalized to *Ubiquitin-C* mRNA (n = 4). (N) Linear regression analysis between *LMO3* and *HSD11B1* mRNA expression in obese human VAT. p values were obtained from regression analysis (n = 45). (O) RNA profiling of transfected hASCs derived from matched human SAT and VAT treated with DMSO or HC for 24 hr. Genes are represented as lines and are normalized mean fluorescence pooled from n = 3 for each treatment group. A summary is shown in Table S4. All error bars represent means ± SEM. p values: ns, not significant; ^∗^p < 0.05, ^∗∗^p < 0.001, and ^∗∗∗^p < 0.0001. See also Figure S1 and Table S1, Table S2, Table S3, Table S4, Table S5, and Table S6.

One of the genes emerging from our screen of the hASC transcriptome in response to Dexamethasone (Dex; full target list in Table S1, available online) that fulfilled our selection criteria, (1) high adipocyte expression and (2) lack of known function in adipogenesis, was the adaptor protein LMO3 (Figure 1A, verified by quantitative PCR in Figure S1A). Several natural as well as synthetic GR ligands, including Dex, hydrocortisone (HC), corticosterone (CC), prednisone (dehydrocortisone) (PRD), and the fluorinated steroids clobetazol (CBTZ) and fluticazone (FLTZ), all potently induced *LMO3* messenger RNA (mRNA), an effect that was blunted by the GR antagonist RU486, suggesting a role of the GR for *LMO3* induction (Figures 1B and S1B). We further silenced the GR to confirm our results utilizing RU486. Transfection of hASCs with a GR-specific small interfering RNA (siRNA), or siGR, resulted in efficient silencing of GR mRNA and protein (Figures 1C and 1D), in contrast to control siRNA (siCtrl). Importantly, siCtrl-transfected hASCs displayed a robust induction of *LMO3* mRNA expression upon Dex treatment, and no such induction was observed upon GR silencing (Figure 1E). To further determine whether GCs upregulate *LMO3* via the GR, we performed transient transfection studies with a luciferase reporter construct of the *LMO3* promoter. GR cotransfection resulted in an approximately 2.5-fold activation of *LMO3* promoter luciferase activity, further enhanced upon treatment with GR ligand Dex. Importantly, *LMO3* promoter activity was blocked when 293FT cells were cotreated with RU486 (Figure 1F). Of note, Dex failed to induce *Lmo3* expression in murine adipose stromal cells (mASC) and 3T3-L1 preadipocytes (Figure S1C).

The promoter regions of GR target genes typically contain one or more GC response elements (GREs), defined by the canonical sequence AGAACAnnnTGTTCT. Many variations of the consensus sequence are possible, including GRE half sites, which are sufficient to generate specific GR binding (van der Laan and Meijer, 2008). Bioinformatic analysis of the proximal promoter region of human *LMO3* revealed two putative GRE half sites, defined by the sequence _−930_TGTTTC_−924_ (GRE1) and _−742_AAAACA_−736_ (GRE2) (Figure 1G). Deletion constructs were created to identify which potential GR-binding sites were involved in transactivation from the *LMO3* promoter reporter construct. Loss of both GRE1 and GRE2 resulted in diminished transactivation, while constructs containing solely GRE2 or both GREs showed potent transactivation (Figure 1G). Dex failed to induce *Lmo3* expression in mouse-derived adipocytes and bioinformatic analysis revealed lack of GRE1 conservation in the mouse *Lmo3* promoter. Therefore, we verified the lack of *Lmo3* expression by exchanging the human with the nonconserved murine GRE1 site (Figure 1G). Again, Dex significantly increased luciferase reporter activity of the original human promoter plasmid (pLMO3-Luc-Hs.), an effect markedly blunted in the luciferase reporter plasmid harboring the murine GRE1 site (pLMO3-Luc-Hs.>Mm.; Figure 1H).

As endogenous GCs need to be activated by 11βHSD1, we tested whether *LMO3* induction is dependent on this mechanism by blocking 11βHSD1 activity with the pharmacologic inhibitor carbenoxolone. This not only prevented PRD-induced *LMO3* expression, but also reduced differentiation into mature adipocytes (Figures 1I and S1D). To elucidate whether LMO3 feeds back into *HSD11B1* expression levels, we silenced *LMO3* in hASCs. Significantly reduced *HSD11B1* expression was observed (Figure 1J), suggesting a positive functional correlation between LMO3 and 11βHSD1 activity, thus comprising partially the permissive role of GCs for adipogenesis. To identify components of the adipogenic cocktail able to induce *LMO3* expression, we assessed the individual components during the early stages of differentiation (24–48 hr). Among the individual components, solely GR ligands Dex and HC, significantly induced *LMO3* in both hASCs and Simpson Golabi Behmel syndrome (SGBS) cells, that are biochemically and functionally similar to human adipocytes (Wabitsch et al., 2001) (Figures 1K and S1E). Again, cotreatment with RU486 potently blocked *LMO3* expression in hASCs (Figure 1K).

Interestingly, paired SAT and VAT samples of 55 obese study participants (Todoric et al., 2011) (Table S2) revealed an increased expression of *LMO3* in VAT compared to SAT, a finding in line with our observation that GCs induce *LMO3* in human fat cells (Figure 1L). To consider the possibility that macrophages present in VAT of obese individuals could account for increased VAT *LMO3* expression, we measured the proportional contribution from different cell types present in VAT. Analysis of a previously published cohort of subfractionated VAT obtained by cell sorting (Zeyda et al., 2007) confirmed the highest *LMO3* mRNA expression in mature adipocytes compared with other cell types including macrophages (Figure 1M). To account for *LMO3* expression arising from CD68-expressing adipose tissue macrophages, we repeated our human paired SAT and VAT comparison on a subset of obese nondiabetic study participants that displayed adipose *CD68* mRNA levels comparable to samples obtained from lean control subjects (Table S3). Importantly, this approach further validated our finding obtained from the entire study cohort and clearly showed that VAT displays substantial higher *LMO3* levels than SAT (Figure S1F). Furthermore, no correlation between *LMO3* and *CD68* mRNA levels was found in the “obese CD68_LOW_” subcohort (Figure S1G). In contrast to cell culture models of adipocyte differentiation in which GCs are added at the beginning of differentiation, white adipose tissue (WAT) 11βHSD1 activity in vivo provides continuous exposure of GCs. Thus, we investigated whether *LMO3* and *HSD11B1* mRNA expression correlate in vivo in WAT and found a strong positive correlation in VAT, but not SAT, in obese subjects (Figure 1N), irrespective of *CD68* expression (Figure S1H and Table S3). To explore whether LMO3 *functionally* affects VAT upon GC treatment, we performed gene expression profiling on LMO3-silenced hASCs isolated from matched SAT and VAT from several donors (Figures 1O and S1J–S1L and Table S4). This analysis revealed (1) higher basal *LMO3* levels in VAT- as compared with SAT-derived preadipocytes (Figure S1I), (2) impaired HC-mediated gene induction in LMO3-siRNA treated VAT cells affecting 21% (181 genes) of all HC-responsive genes (Figure S1J), (3) importantly, no differences in SAT-derived hASCs upon silencing of LMO3 (Figure 1O left versus right panel), and (4) LMO3-dependent and VAT-specific enrichment of genes regulating cell differentiation, communication, and adhesion, as well as signal transduction. Of note, several of these LMO3-enriched genes are well known GC targets, such as inhibitor of growth family, member 2 (*ING2*), TBC1 domain family, member 2B (*TBC1D2B*), tumor necrosis factor (ligand) superfamily, member 10 (*TNFSF10*), C-X-C motif chemokine 5 (*CXCL5*), and ecto nucleotide pyrophosphatase/phosphodiesterase 2 (*ENPP2*) (Bujalska et al., 2006; Lu et al., 2007; Viguerie et al., 2012) (Figures S1K and S1L). These data suggest that the (ligand-bound) GR is critical for GC-mediated *LMO3* expression. Further, LMO3 is a specific target and amplifier of GC action in human adipocytes displaying a VI fat pattern, i.e., high levels of VI expression and close linkage with *HSD11B1* levels. Thus, LMO3 is modulating GC-triggered responses of human (pre)adipocytes in a depot-, i.e., VAT-, specific manner.

### LMO3 Is Induced in Human Adipogenesis

**Figure 2:**
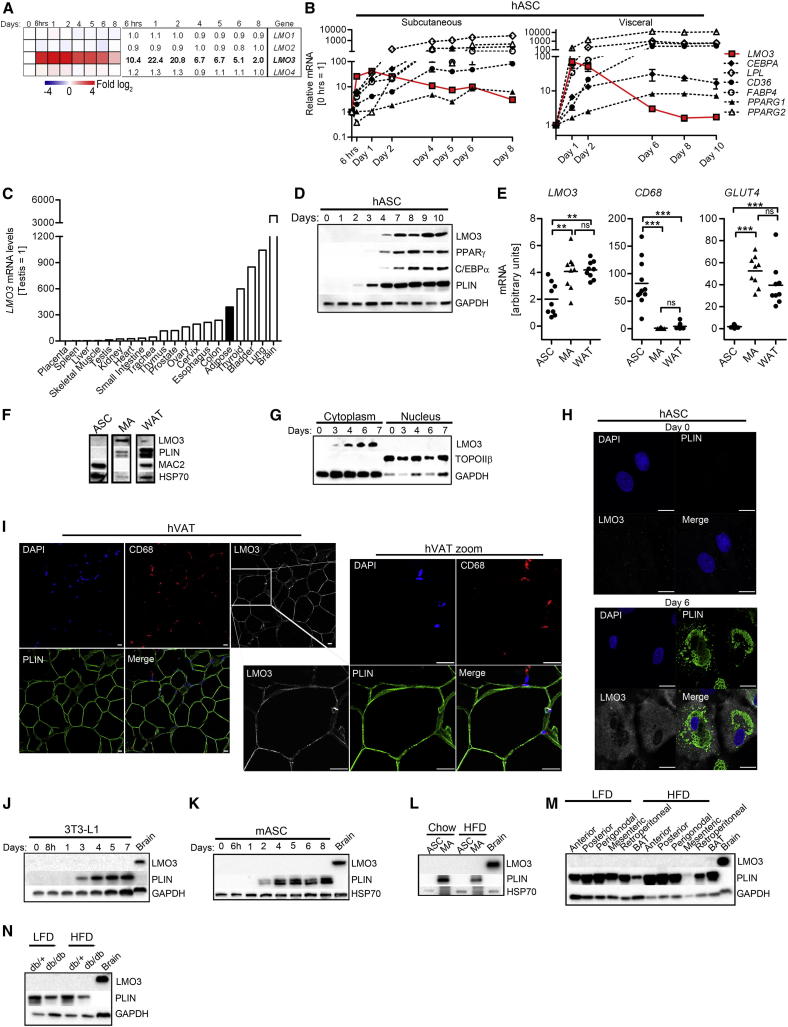
Human, Not Mouse, LMO3 Is Induced during Adipocyte Differentiation (A) *LMO1-4* mRNA expression based on DNA microarray expression profiling. Fold change compared to day 0 of differentiation is shown. (B) mRNA was determined in differentiating hASCs isolated from SAT or VAT as the mean fold change ± SEM (n = 3). (C) *LMO3* mRNA tissue distribution in humans. Each tissue is pooled from three donors. (D) Protein expression throughout differentiation in SAT-isolated hASCs. (E and F) mRNA and (F) protein (F) expression in human SAT cell fractions (n = 9). (G) Protein expression in nuclear and cytosolic fractions in differentiating SAT-derived hASCs. (H and I) Images are representative of multiple donors. Scale bars represent 20 μm. Stainings in differentiating SAT-derived hASCs (H) and human VAT sections (I) are shown. (J–N) Determination of protein expression in differentiating murine 3T3-L1s (J), primary murine adipose stromal cells (mASCs) isolated from C57BL/6J perigonadal pads (n = 5) (K and I), the indicated cell subpopulations from C57Bl/6 perigonadal pads (n = 5) (L), WAT depots from C57BL/6J mice (n = 5) (M), and perigonadal pads from db/db mice and littermate controls (db/+) (n = 3–4) (N). All error bars represent the means ± SEM. p values: ns, not significant; ^∗∗^p < 0.001 and ^∗∗∗^p < 0.0001. See also Figure S2.

In order to better understand the biological function of LMO3 in adipose tissue/adipocytes, we examined whether LMO3 is regulated during fat-cell formation in vivo and in vitro. DNA microarray analysis of hASCs treated with an adipogenic cocktail revealed strong induction of *LMO3*, whereas other LMO family members were unaffected (Figure 2A). The *LMO3* expression pattern was determined in hASCs isolated from SAT, but also holds true for VAT-derived hASCs and for SGBS preadipocytes, when induced to differentiate into mature adipocytes (Figures 2B and S2A). Furthermore, mature adipocyte markers *ADIPOQ*, *LPL*, *PLIN*, *CD36*, and *FABP4* and the transcriptional master regulators *PPARG* and *CEBPA* confirmed successful generation of mature adipocytes (Figures 2B, S2A, and S2B). Adipose tissue *LMO3* mRNA expression was among the top 25% of all human tissues examined (Figure 2C). Importantly, LMO3 protein levels could be detected simultaneously with the master regulators PPARγ and CEBPα in hASCs induced to differentiate into mature adipocytes (Figure 2D). Fractionation of WAT allowed us to identify mature adipocytes as the dominant site of *LMO3* mRNA and protein expression within WAT (Figures 2E and 2F). Thus, we show in patient fat biopsies and in two well-established in vitro models that LMO3 expression increases throughout adipogenesis. To determine subcellular LMO3 protein expression during adipogenesis, we isolated cytoplasmic and nuclear protein extracts throughout differentiation of hASCs into mature adipocytes. We demonstrate a strong increase in LMO3 expression from day 3 to day 7 that was detectable in cytoplasmic but not nuclear fractions (Figure 2G). Restriction of LMO3 expression to the cytoplasm was further corroborated by confocal immunofluorescence analysis of hASCs undergoing differentiation (Figures 2H and S2C) showing no LMO3 signal in preadipocytes (day 0) but strong cytoplasmic expression in perilipin (PLIN)-positive adipocytes (day 6). Occasionally, WAT-resident CD68-positive macrophages also stained positive for LMO3 (Figure 2I and S2D). Perhaps most intriguing, LMO3 induction was specific for human adipogenesis, as murine adipocyte differentiation was not accompanied by enhanced *Lmo3* expression. Lack of *Lmo3* expression was confirmed in (1) WAT of a mouse tissue library (Figure S2E), (2) the murine 3T3-L1 adipocyte cell model throughout differentiation (Figures 2J and S2F), and (3) differentiating murine primary preadipocytes (Figure 2K), as well as in (4) ASCs and mature adipocytes isolated from chow-fed or high-fat diet (HFD)-challenged mice (Figures 2L and S2G), (5) distinct SC and VI WAT depots and BAT of mice fed a low-fat diet (LFD) or HFD (Figures 2M and S2H), and (6) WAT obtained from a genetic obesity mouse model (db/db) on both LFD and HFD (Figures 2N and S2I). *Lmo3* expression was highest in the brain and therefore was used as a positive control throughout analysis with murine samples (Figures 2J–2N and S2G–S2I). Thus, LMO3 is a marker of human adipogenesis not applicable for mice.

### LMO3 Promotes Adipogenesis

**Figure 3:**
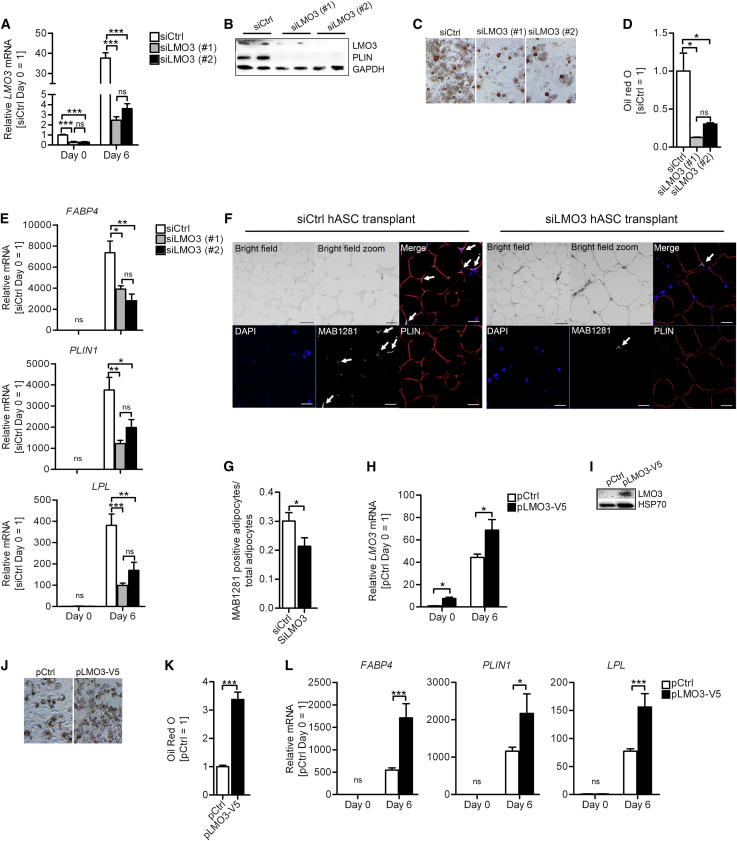
RNA-Interference-Mediated Knockdown of LMO3 Suppresses, whereas Overexpression of LMO3 Promotes, Adipogenesis (A, D, E, and G) Comparisons of control (siCtrl)- versus LMO3-silenced (siLMO3)-transfected cells. (A and B) mRNA (A) and protein (B) verification of LMO3 silencing on the indicated days (blots day 6) of differentiation in transfected hASCs. (C) Mature adipocytes (differentiation day 10) stained with oil red O. Microscopic views, magnifications 10×. (D) Quantification of oil red O staining in (C) (n = 3). (E) RT-PCR analysis in transfected hASCs. (n = 3). (F) Representative immunofluorescent staining of xenotransplanted SCID mice. From left to right: top, bright-field morphology of the transplant sections (the scale bar represents 50 μm), zoomed bright-field image (the scale bar represents 20 μm), and immunofluorescent merge; bottom, DAPI (blue), MAB1281 (gray with arrows), and perilipin (red) (immunofluorescent panels, the scale bar represents 20 μM). (G) Quantification of xenotransplanted SCID mice stainings. (H, K, and L) Comparisons of control (pCtrl)- or LMO3 (pLMO3-V5)-transfected cells. (H and I) mRNA (H) and protein (I) verification of LMO3 overexpressing transfected hASCs on the indicated days. (J) Oil red O stain of overexpressing hASCs (differentiation day 10). Shown are microscopic views, magnifications 10× (n = 3). (K) Quantification of oil red O stain in (J) (n = 3). (L) RT-PCR analysis in pCtrl or pLMO3-V5 transfected hASCs. (n = 3). All error bars represent the means ± SEM. p values: ns, not significant; ^∗^p < 0.05, ^∗∗^p < 0.001, and ^∗∗∗^p < 0.0001. See also Figure S3.

To clarify the role of endogenous LMO3 in adipocyte differentiation, we silenced the expression of *LMO3* in subconfluent hASCs using siRNA and induced differentiation. To reduce the risk of potential off-target effects, we applied two different siRNA oligonucleotides (referred to as siLMO3 #1 and siLMO3 #2) targeting different *LMO3* exons. Figures 3A and 3B show efficient LMO3 mRNA and protein knockdown in hASCs. Cells with reduced LMO3 demonstrated diminished adipogenic potential, including less lipid accumulation (Figures 3C and 3D) and diminished expression of adipocyte marker genes, including *FABP4*, *PLIN1*, and *LPL* (Figures 3B [perilipin blot] and 3E). Importantly, neither cell viability nor cell proliferation varied between siCtrl- and siLMO3-treated hASCs under the experimental conditions for adipocyte differentiation (Figures S3A and B). We additionally verified the hASC requirement of GCs to potentiate the adipogenic program by excluding GCs. This resulted in almost complete lack of mature adipocyte formation also in LMO3-silenced hASCs, as expected (Figure S3C), demonstrating that GCs are indeed necessary to induce adipogenesis in human preadipocytes. To study the role of LMO3 in regulation of adipogenesis in vivo, we subdermally administered control- or LMO3-silenced hASCs into severe combined immunodeficient (SCID) mice. The transplants were collected and histologically examined after 6 weeks. We confirmed the human origin of the collected cells by immunofluorescent staining with MAB1281, a human-specific nuclear antibody that does not stain mouse nuclei (Figures 3F and S3D). Mature adipocytes also stained positive for the adipocyte marker perilipin. We observed a significantly higher proportion of hASC-derived mature adipocytes in tissues collected from animals that were transplanted with siCtrl-treated hASCs as compared to mice transplanted with siLMO3-transfected hASCs (Figure 3G), underlining strong proadipogenic effects of LMO3 also in vivo.

Next, we employed LMO3 gain-of-function studies to minimize the risk that the siLMO3-triggered reduction of adipocyte differentiation is an unspecific side effect due to our experimental manipulations interfering with a highly coordinated, and thus sensitive, cellular program. *LMO3* mRNA and protein levels were significantly increased in LMO3 cells transfected with a LMO3 expression plasmid relative to cells transfected with a control plasmid (Figures 3H and 3I, pCtrl versus pLMO3-V5). In accordance with our loss-of-function data elucidating LMO3 as a proadipogenic mediator, overexpression of LMO3 in these cells significantly enhanced adipogenesis, shown by increased oil red O staining of neutral lipids (Figures 3J and 3K) and significant overexpression of the adipocyte markers *FABP4*, *LPL*, and *PLIN1* (Figure 3L). As LMO3 overexpression promotes adipogenesis and expression of genes facilitating lipid accumulation in hASCs, we investigated whether visceral *LMO3* expression is linked to body mass index (BMI), waist circumference, or HOMA-IR in our nondiabetic lean and obese patient cohorts (Tables S5 and S6). Interestingly, and despite the fact that *HSD11B1* levels were robustly increased in the VAT of obese study subjects displaying high HOMA-IR (Table S5), no such difference could be found for *LMO3* expression.

Of note, siRNA mediated knockdown of mouse *Lmo3* failed to interfere with adipogenesis in 3T3-L1 cells (Figures S3E and S3F). Interestingly, when *Lmo3* was overexpressed, it exerted the phenotype observed in hASCs, i.e., enhanced adipogenesis (Figures S3G and S3H), implying that murine cells can utilize *Lmo3*, but that due to lack of conservation in the GRE1 site, it is not inducible. Thus, LMO3 is a prerequisite to unveil the full adipogenic potential of human preadipocytes.

### LMO3 Boosts a Proadipogenic PPARγ Program

**Figure 4:**
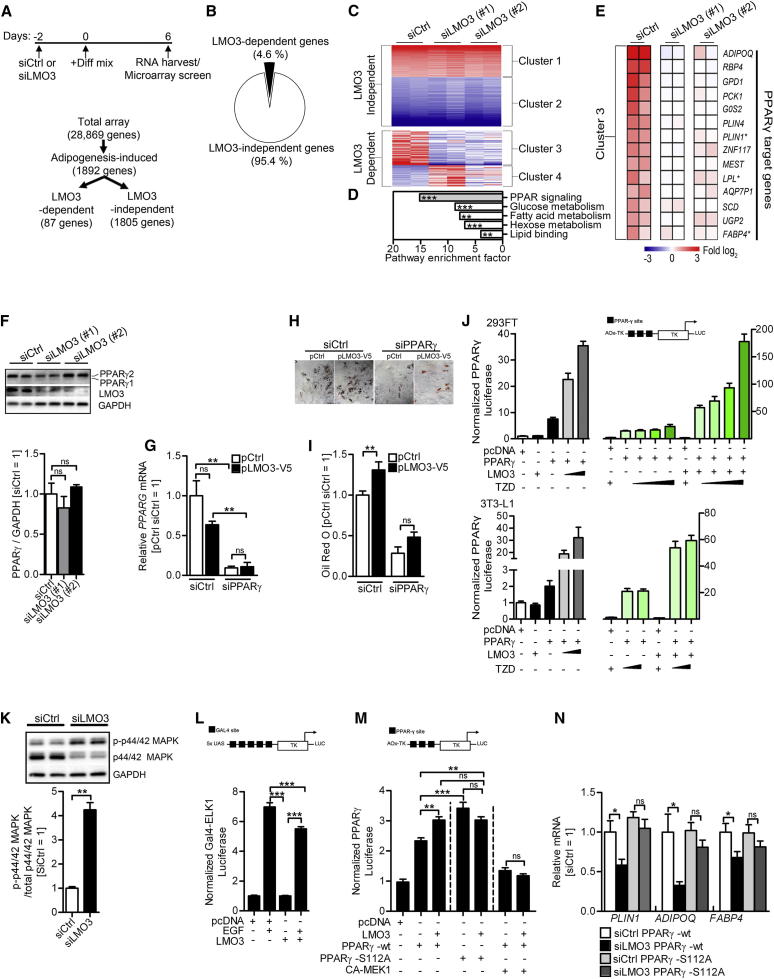
LMO3 Promotes Adipogenesis via Increasing PPARγ Tone (A) Flow chart with experimental DNA microarray setup and gene selection process. (B) Schematic pie chart of LMO3-dependent and -independent genes. (C) Clusters of genes from the microarray screen. Red is up- and blue is downregulated genes. (D) Pathway enrichment analysis shown as a *Z* score on LMO3-dependent genes (87 in total) with DAVID and EASE. (E) Heatmap diagram of PPARγ target genes within the LMO3-dependent gene signature. Experiments were performed in duplicates. ^∗^, independent validation shown in Figure 3E. See Table S7 for LMO3-dependent target genes at day 6 of differentiation. (F) PPARγ protein expression on day 6 of differentiation in transfected hASCs. Densitometric evaluation is shown below. (n = 3.) (G) Silenced *PPARG* mRNA verification in transfected hASCs at day 9 of differentiation. (H) Oil red O staining of cells treated as in (G), magnifications 10×. (I) Quantification of (H). pCtrl/siCtrl-transfected cells are set to 1 for comparison. (J) LMO3 enhances PPARγ activation of a luciferase reporter driven by minimal PPAR-responsive elements (3X-PPRE) (“pAOx-TK”) in 293FT (top) and 3T3-L1 (bottom) cells stimulated for 24 hr with DMSO or TZD. pcDNA with DMSO, set to 1 for comparison. (n = 3.) (K) Transfected and day 6 differentiated hASCs were serum starved overnight followed by 1 hr 40% fetal bovine serum (FBS). The top panels are representative blots. The bottom panels show densitometric evaluation. (L) Luciferase reporter activity analysis of GAL-ELK-1 constructs in 293FT cells. The construct is depicted above. (M) 293FT cells transfected with wild-type (WT) PPARγ or S112A mutant were analyzed for TZD-induced reporter activity using PPRE-luciferase (as in J) in the presence or absence of LMO3 (n = 3). (N) mRNA of mature adipocyte markers in hASCs with WT PPARγ transfected with either siCtrl or siLMO3 oligo (white and black bars) and/or additionally cotransfected with mutated PPARγ S112A plasmid (gray bars). RNA was isolated from cells on day 8 of differentiation. All error bars represent the means ± SEM. p values: ns, not significant; ^∗^p < 0.05, ^∗∗^p < 0.001, and ^∗∗∗^p < 0.0001. See also Figure S3 and Table S7.

We next sought to investigate the mechanism by which LMO3 promotes adipogenesis. Thus, we profiled genome-wide expression changes that occur in response to the adipogenic cocktail, comparing patterns between siCtrl- or siLMO3-treated human preadipocytes on day 6 of differentiation, integrating both primary and secondary effects of LMO3. Figure 4A shows 1,892 genes from adipogenesis-induced preadipocytes that displayed at least a 1.5-fold expression change relative to day 0 and were therefore selected as adipogenesis-induced genes. Approximately 4.6% of the adipogenic gene signature was affected by *LMO3* knockdown with two independent siRNA oligonucleotides targeting *LMO3* mRNA (Figure 4B). Hierarchical clustering partitioned the 1,892-adipogenesis-induced genes into LMO3-independent clusters 1 and 2 or LMO3-dependent clusters 3 and 4 (Figure 4C and Table S7). Pathway enrichment analysis of LMO3-dependent cluster 3 revealed a highly significant enrichment for PPAR signaling (Figure 4D). In agreement with the pathway enrichment results, inspection of cluster 3 showed that silencing of *LMO3* (siLMO3) diminished expression levels of several known PPARγ target genes in hASCs (Figure 4E). Therefore, we tested whether LMO3 modulates PPARγ expression and/or activity. Western blotting of siCtrl- and siLMO3-treated hASCs suggested a slight but nonsignificant impact on PPARγ protein expression (Figure 4F). We further investigated whether PPARγ activity is needed for the proadipogenic effects of endogenous LMO3 on lipid accumulation. We silenced *PPARG* (siPPARγ) in hASCs overexpressing LMO3 (pLMO3-V5) and evaluated lipid accumulation by oil red O staining in differentiating hASCs 8 days later (Figures 4G–4I). As shown above (Figures 3J and 3K), overexpression of LMO3 increased lipid accumulation (Figures 4H and 4I, left panels). Of note, silencing of the adipogenic master regulator *PPARG* (siPPARγ) abolished the proadipogenic effect of LMO3, suggesting that LMO3 acts upstream of PPARγ (Figures 4H and 4I, right panels). We next determined whether LMO3 is able to modulate the transcriptional activity of PPARγ. We performed transfection assays in 293FT or 3T3-L1 cells with a reporter driven by isolated PPAR response elements (PPREs). As expected, cotransfected *PPARG* resulted in an increase in luciferase activity, in part because of the ligand-independent activation function in its amino terminus. Troglitazone (TZD) treatment further enhanced *PPARG* activity in a dose-dependent manner. Cotransfection with increasing amounts of LMO3 expression plasmid increased *PPARG* activity, which was further enhanced in the presence of TZD (Figure 4J). Thus, LMO3 drives adipogenesis through increasing PPARγ tone.

A critical step required during adipogenesis is the downregulation of mitogen-activated protein kinase extracellular signal-regulated kinases (MAPK-ERKs) mediated phosphorylation at serine 112 (S112) in the N-terminal region of PPARγ, which blocks PPARγ to activate the full proadipogenic gene program (Adams et al., 1997; Camp and Tafuri, 1997; Hu et al., 1996; Shao et al., 1998). Interestingly, we found that loss of LMO3 resulted in increased serum-induced ERK1/2 phosphorylation (p-p44/42 MAPK; Figure 4K), further supporting the observation that LMO3 acts upstream of PPARγ. To test whether LMO3 could directly inhibit ERK-dependent signaling, we performed transient transfection assays using reporter plasmids that read out ERK-dependent activation of the transcription factor ELK1. Cotransfection of a LMO3 expression vector diminished the ability of EGF to activate the ELK1 reporter (Figure 4L). Importantly, compared with control cells, the LMO3 knockdown cells not only showed increased ERK1/2 phosphorylation but also 1.7-fold increased phosphorylation levels of endogenous PPARγ at S112 (Figure S3I). Next, we addressed whether p-S112 is involved in LMO3-mediated effects by making use of a mutant PPARγ-S112A, which renders PPARγ refractory to p-S112-mediated inactivation (Camp and Tafuri, 1997; Hu et al., 1996; Shao et al., 1998). We initially tested whether LMO3 increases TZD-mediated PPARγ activity via S112 when a reporter driven by isolated PPREs (AOx-TK) was used. As reported, mutant PPARγ-S112A displayed increased PPARγ activity (Adams et al., 1997). Again, LMO3 cotransfection boosted PPARγ promoter activity. However, and in sharp contrast, LMO3 was unable to further increase the transcriptional activity of mutated PPARγ-S112A (Figure 4M). Similarly, cotransfection with an activated allele of MEK, the upstream kinase of ERK1/2, prevented the stimulating effect of LMO3 on PPARγ activity (Figure 4M). In line with our previous results, LMO3 knockdown reduced adipogenesis and the expression of PPARγ target genes in hASCs expressing wild-type PPARγ (Figures 4N). However, and importantly, these LMO3-dependent effects were lost in hASCs transfected with the mutated form of S112 PPARγ (PPARγ-S112A) or the presence of a constitutively active MEK (Figure 4N). Thus, interference with MAPK-ERK phosphorylation of PPARγ is one possible mechanism by which LMO3 regulates human adipocyte differentiation.

## Discussion

Obesity is associated with many metabolic consequences, where VI fat accumulation produces a greater risk of diabetes, dyslipidemia, and accelerated atherosclerosis (Kissebah et al., 1982; Wajchenberg, 2000). In this study, we aimed to identify GC target genes involved in the differentiation of human adipocytes, on the basis of (1) a hitherto unknown function in adipocyte biology, (2) a robust induction in human adipocyte differentiation models, and (3) the potential to act upstream of the adipogenic master regulator PPARγ. Using these criteria, we identified among the top-most regulated genes *LMO3*.

*LMO3* promoter studies, GR silencing, GR antagonist RU486, and several natural and synthetic GCs showed that LMO3 is a direct GR target gene. Interestingly, we found that *LMO3* is not only induced by GCs and HSD11B1, but is also part of a positive feedback loop enhancing GC action on adipocytes, a finding paralleled by our data showing tightly correlated *LMO3* and *HSD11B1* levels in human VAT but not SAT. Overexpression of *LMO3* in hASCs enhanced adipogenesis and was reflected by enhanced adipogenic gene expression and enhanced lipid accumulation. Knockdown of *LMO3* in hASCs suppressed fat differentiation both in vitro and in vivo. This collectively renders LMO3 as an essential factor linking extrinsic factors (GCs) with specific molecular mediators, resulting in progressed adipogenesis. To better understand the role of LMO3 in human depot-specific responses to GCs, we silenced *LMO3* expression in hASCs from matched SAT and VAT. Upon GC treatment, an LMO3-dependent gene expression signature was observed solely in VAT- but not SAT-derived preadipocytes, a finding that may be related—at least in part—to the higher basal *LMO3* and *HSD11B1* expression in VAT-derived preadipocytes. Of note, both fat depots are responsive to the actions of LMO3 in vitro; however, in vivo measurements and SAT/VAT comparisons revealed a clear preference of VI preadipocytes for LMO3-dependent GC action. Some of the LMO3-dependent genes expressed in VI but not SC preadipocytes were *ENPP2*, *TNFSF10* or *TBC1* domain family member 2B (TBC1D2B) potentially having direct influence on (VI) fat-cell growth or metabolism. The lysophospholipase ENPP2 and its product, lysophosphatidic acid, have established effects on preadipocyte proliferation and fat-tissue expansion, and its expression is enhanced in a depot-specific manner in obese/insulin-resistant individuals (Rancoule et al., 2012), whereas the secreted protein TNFSF10 regulates adipocyte metabolism through cleavage of PPARγ (Keuper et al., 2013). The GC-induced signaling nexus TBC1D2B may enhance insulin signaling in a manner reported for its close paralogues, TBC1D1, TBC1D3, and TBC1D4 (Pehmøller et al., 2012; Wainszelbaum et al., 2012) in a LMO3-dependent manner in VAT-, but not SAT-, derived preadipocytes, adding to adipose depot-specific actions of insulin (Hazlehurst et al., 2013). We now add LMO3 to the growing list of developmental regulators controlling (depot-specific) adipocyte differentiation (Macotela et al., 2012).

Several observations led us to suggest that LMO3 enhances adipogenesis via PPARγ: (1) the most significant category of genes suppressed after *LMO3* silencing in differentiating hASCs was PPARγ target genes, (2) RNA-interference-mediated silencing of *PPARG* during hASC differentiation abrogated LMO3-enhanced lipid accumulation, and, most importantly, (3) LMO3 overexpression enhanced PPARγ transcriptional activity in two cell models. These observations raised the question of how cytoplasmic LMO3 enhances primarily nuclear PPARγ activity. One potential mechanism is the well studied inhibition of PPARγ activity by ERK1/2-mediated phosphorylation of PPARγ at serine 112 (Adams et al., 1997; Hu et al., 1996; Shao et al., 1998). Interestingly, we observed that LMO3-deficient cells displayed an increase in the amount of p-S112 PPARγ and p-ERK1/2, while LMO3-overexpressing cells display reduced ERK1/2 pathway activity. Importantly, we were also able to reverse the LMO3 knockdown-based phenotype by PPARγ -S112A transfection, a mutation that has been described to render PPARγ insensitive to pS112-inhibition (Adams et al., 1997; Hu et al., 1996). Taken together, this supports our hypothesis that LMO3 targeting of PPARγ at serine 112 (via ERK1/2) represents a major determinant altering adipocyte gene expression. Interestingly, a similar mechanism, i.e., lack of catalytic activity and negative modulation of ERK1/2, has been reported to underlie the proadipogenic effects of the cytoplasmic downstream of tyrosine kinase-1 (DOK1) (Hosooka et al., 2008). Indeed, the LIM domains of LMO3 lack intrinsic catalytic (i.e., phosphatase) activity. However, as for DOK1, LIM proteins mediate many biological processes acting as a docking site for the assembly of multiprotein complexes (Kadrmas and Beckerle, 2004; Zheng and Zhao, 2007). Among others, potential LMO3 interaction partners include ERK activators MAPK kinases (MAPKK/MEK) (Burgermeister et al., 2007), ERK1/2 themselves, (Adams et al., 1997; Hu et al., 1996), phosphatases (Hinds et al., 2011), and the signaling adaptor DOK1 (Hosooka et al., 2008).

The 11βHSD1/LMO3/ PPARγ module provides differentiating human (pre)adipocytes with a molecular switch, enabling the cells to fine-tune their response to circulating GCs. *11βHSD1* is expressed at high levels in VI fat depots (Bujalska et al., 1997, 2008; Morton, 2010; Walker and Andrew, 2006), and LMO3 is also found at higher levels in VAT as compared to SAT. Therefore, it is proposed that GCs drive human *LMO3* expression and thus its proadipogenic activities in a depot-specific, VI manner. In such a scenario, low 11βHSD1 activities or its inhibition by pharmacologic targeting will result in (1) reduced VI LMO3 levels, (2) reduced PPARγ transcriptional activities, and, consequently (3) reduced adipogenesis, specifically in VI fat.

An appealing question is why LMO3 can be averted in mouse adipocytes and why there is no obvious functional consequence. This is especially interesting, since ectopically expressed *Lmo3* in murine 3T3-L1 cells enabled replication of the phenotype observed in differentiated hASCs (i.e., enhanced adipogenesis). As a direct consequence, *Lmo3*-dependent fine-tuning in mice does not apply, not because mice cannot utilize *Lmo3* to enhance adipogenesis, but because the critical GC induction site GRE1 is mutated in the mouse genome. Thus, LMO3 might represent a mechanism by which—in contrast to mice—humans can adapt and modulate the activity of the key adipogenic master regulator PPARγ. Although the basic molecular mechanisms of fat-cell development are identical in rodents and humans—as is the intact response of murine cells to reintroduced Lmo3—many of the observed species-specific attributes likely stem from when (and where) the products of the genes are made (Wilson et al., 2008) so that the timing of preadipocyte recruitment and adipocyte differentiation is accessible to more subtle fine-tuning mechanisms.

Interestingly, as opposed to *HSD11B1*, no correlations were observed between *LMO3* and BMI or HOMA-IR in our study participants. However, the missing link of *LMO3* expression with our metabolic parameters needs to be interpreted with caution since several circumstances may have blurred a potential relationship (specifically, considering observed *HSD11B1* correlations with *LMO3* in VAT of obese subjects; Table S6). Among others, we cannot exclude the possibility that nonfat cells in VAT may obscure the association of LMO3 with metabolic parameters. Also, we cannot exclude that we missed the best time point to collect our fat biopsies, i.e., it might have been too late in the course of fat-cell recruitment and expansion, especially in our obese study cohort. Further, GC circadian rhythms may have masked pre-existing differences in LMO3 expression (Peckett et al., 2011). Further studies with highly standardized measurements of circadian systemic and adipose GC levels are needed to relate LMO3 with states of obesity and diabetes.

**Figure 5:**
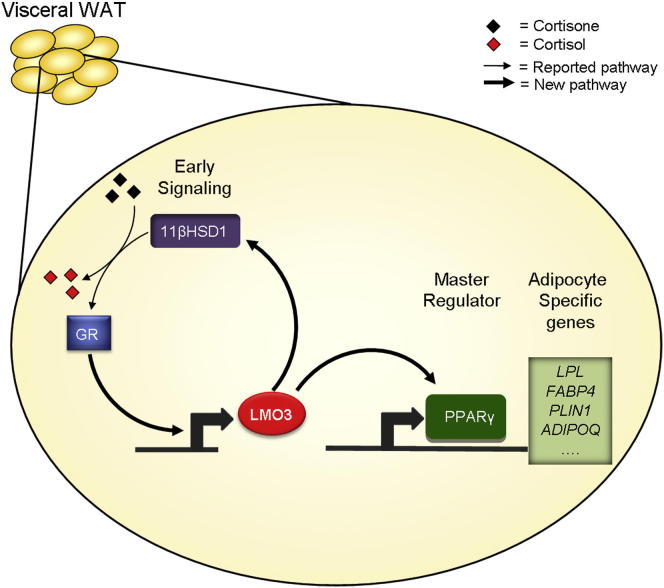
LMO3 Is a Human Driver of Adipogenesis Schematic model of the pathways controlling differentiation in hASCs.

Our current study (summarized in Figure 5) added LMO3 as a proadipogenic protein and suggests that LMO3 modulates human adipocyte differentiation by acting on PPARγ, between the early and late phase of adipocyte differentiation (Farmer, 2006). Our data also help to explain, at least in part, the long-known but ill-defined effect of GCs on VAT. Finally, we propose that the preferential expression in VAT, the GC responsiveness, and the functional location upstream of PPARγ make LMO3 an attractive target to interfere with human adipocyte differentiation in a depot-specific, therapeutically relevant manner.

## Experimental Procedures

### Human Samples and Clinical Parameters

Study subjects included 55 obese patients and seven nonobese controls that underwent weight-reducing surgery or elective surgical procedures such as cholecystectomy. Participants were included if they had fasting plasma glucose levels <7.0 mmol/liter, no history of diabetes or use of blood-glucose-lowering medications, no weight changes >3% during the previous 2 months, and C-reactive protein (CRP) levels <20 mg/liter. All study subjects provided informed consent, and study protocols were approved by the local Ethics Committee. Tissue biopsies from SAT and VAT, obtained during surgery, were stored at −80°C until further processing. Plasma glucose, insulin, and CRP were determined as described (Todoric et al., 2011).

### Isolation of Preadipocytes

Human SAT was obtained from healthy individuals undergoing lipoaspiration. A total of 47 donors (female, n = 34; male, n = 13) were used throughout the study; Total age was 44.02 ± 14.7 years (female, 46.47 ± 14.2; male, 37.62 ± 14.5), and total mean BMI was 25.08 ± 4.3 (female, 24.87 ± 4.5; male, 24.36 ± 3.3). Matched SAT and VAT was obtained from three of the above donors undergoing elective abdominal surgery. This study was approved by the Medical University of Vienna’s ethics committee and the General Hospital of Vienna (EK no. 1115/2010). All subjects gave written informed consent prior to taking part in the study.

### Mouse Studies

Mice were purchased from Charles River Laboratories. Male C57BL/6J, BKS.Cg-Dock7^m^+/+ Lepr^db^/J diabetic (db/db) and nondiabetic (db/+) littermates were used as detailed in the Supplemental Experimental Procedures.

### Human and Murine Adipocyte Differentiation

Two-day-postconfluent ASCs were induced to differentiate for 10 to 13 days with (the medium used is referred to as “full mix” in the text) Dulbecco’s modified Eagle’s medium (DMEM)/Ham’s F12, 10% FBS, 33 μM biotin, 17 μM pantothenic acid, 1 nM triiodothyronine (T3), 870 nM human insulin, 5 μM TZD, and 1 μg/ml transferrin, and for the first 3 days 1 μM Dex and 500 μM isobutyl-methylxanthine (IBMX) were included (all from Sigma). Two-day-postconfluent 3T3-L1 cells were differentiated with DMEM, 10% CS, 870 nM insulin, 1 μM Dex, and 500 μM IBMX. On day 3 of differentiation, this medium was added excluding IBMX and Dex for remaining differentiation. Additional compounds used were 100 nM HC, 100 nM CC, 1 μM PRD, 5 μM CBTZ, 5 μM FLTZ, 100 μM carbenoxolone, and 1 μM RU486.

### Gene Expression Profiling

Was performed as previously described (Bilban et al., 2009). For extended information on GC target genes and LMO3 targets after GC stimulation, refer to the Supplemental Experimental Procedures.

### Real-Time PCR

Real-time PCR was performed as previously published (Todoric et al., 2011). Primer sequences are listed in the Supplemental Experimental Procedures.

### Adipose Tissue Fractionation

Human ASCs and MA were isolated as described above. ASCs were fractioned by flow cytometry (FACSAria, BD Biosciences) as previously described (Zeyda et al., 2007).

### Luciferase Assays

Luciferase assays were carried out as previously described (Bilban et al., 2009) and as detailed in the Supplemental Experimental Procedures.

### hASC Transfection

siRNA (100 nmol/liter) (listed in the Supplemental Experimental Procedures) (all Invitrogen) or plasmids (1 μg) were delivered into hASCs (6 × 10^5^) by Amaxa nucleofection (Lonza Bioscience) according to manufacturer‘s recommendations. Cells were utilized 48–72 hr after transfection.

### Western Blot Analyses

Western blot analyses were performed as described previously (Bilban et al., 2009).

### Confocal Immunofluorescence Microscopy

All immunofluorescence slides were mounted for imaging with confocal laser scanning microscopy (LSM 700 Carl Zeiss) as detailed in the Supplemental Experimental Procedures.

### SCID Mouse Xenotransplant Model

All procedures were carried out in accordance with the Association for Assessment and Accreditation of Laboratory Animal Care guidelines and the Guide for the Care and Use of Laboratory Animals (US Department of Health and Human Services, National Institutes of Health, publication no. 86–23). All experiments were approved by the ethics committee of the Medical University of Vienna and by the Austrian government committee on animal experimentation. For further information, see the Supplemental Experimental Procedures.

### Statistical Analysis

The significance of differences between means was assessed by two-tailed Student’s t test or analysis of variance (ANOVA) with Bonferroni post test. Differences between human adipose tissue depots were ascertained by ANOVA. Correlations were tested by linear regression. Logarithmic transformations were made if the equal variance and normality assumptions were rejected. All measurements were adjusted for confounding effects as indicated. Error bars are expressed as the mean ± SEM unless otherwise specified. p < 0.05 was considered significant.

## Accession Numbers

Data sets have been deposited in the Gene Expression Omnibus (GEO) archive as series GSE44636.

## Supplemental Information

Document S1. Supplemental Experimental Procedures, Figures S1–S3, and Tables S2, S3, S5, and S6

Table S1. GC Target Genes in hASCs, Identified by DNA Microarray Analysis, Related to Figure 1 hASCs were treated with 1 μM Dex in growth medium, and total RNA was isolated every 24 hr from day 0 up to day 5. mRNA expression profiles were generated with human Gene 1.0 ST Arrays and processed as described in the Experimental Procedures. Genes exhibiting more than a ±3-fold change relative to control treatment (in at least three out of five comparisons) were tabulated.

Table S4. LMO3-Sependent Genes from Matched SAT and VAT after Hydrocortisone Stimulation, Related to Figure 1 siCtrl- or siLMO3-tansfected hASCs derived from matched human SAT and VAT and pooled from n = 3 different donors treated with DMSO or HC for 24 hr. mRNA expression profiles were generated with human Gene 1.0 ST Arrays and processed as described in the Experimental Procedures. First, genes responsive to HC were tabulated if the fold change (HC versus vehicle) was more than ±1.5-fold in siCtrl- or siLMO3-transfected hASCs. Genes were then classified as LMO3 dependent in HC-treated VAT if the fold change was more than ±1.5-fold in siCtrl- versus siLMO3-transfected hASCs.

Table S7. LMO3 Dependent and Independent Target Genes Shown in Clusters 1–4, Related to Figure 4 mRNA profiling of siCtrl- or siLMO3-transfected hASCs differentiated into mature adipocytes. Total RNA was isolated at day 0 and day 6 of differentiation and subjected to DNA microarray analysis with Gene 1.0 ST arrays as described in the Experimental Procedures. Clusters 1 and 2 contain genes responsive to the adipogenic cocktail in siCtrl-treated (control) hASCs with mean ± 1.5-fold expression day 6 versus day 0 of differentiation. Next, from these 1,892 genes we identified LMO3-dependent genes by comparing fold changes (day 6 versus day 0) of siCtrl with LMO3 knockdown (siLMO3 #1 or siLMO3 #2) hASCs. Genes were classified as LMO3 dependent if the fold change was ±1.5-fold different between siCtrl- or siLMO3-transfected hASCs (shown in clusters 3 and 4).
