# Supplementary material for: Human but Not Mouse Adipogenesis Is Critically Dependent on LMO3
Source: Cell Metab. 2013 Jul 2;18(1):62–74. doi: 10.1016/j.cmet.2013.05.020 (PMC3701325; doi:10.1016/j.cmet.2013.05.020)
Supplement: Document S1. Supplemental Experimental Procedures, Figures S1–S3, and Tables S2, S3, S5, and S6 [file mmc1.pdf]

Cell Metabolism, Volume *18*

Supplemental Information

Human but Not Mouse Adipogenesis

Is Critically Dependent on LMO3

Josefine Lindroos, Julia Husa, Gerfried Mitterer, Arvand Haschemi, Sabine Rauscher, Robert Haas, Marion Gröger, Robert Loewe, Norbert Kohrgruber, Klaus F. Schrögendorfer, Gerhard Prager, Harald Beck, J. Andrew Pospisilik, Maximilian Zeyda, Thomas M Stulnig, Wolfgang Patsch, Oswald Wagner, Harald Esterbauer, and Martin Bilban

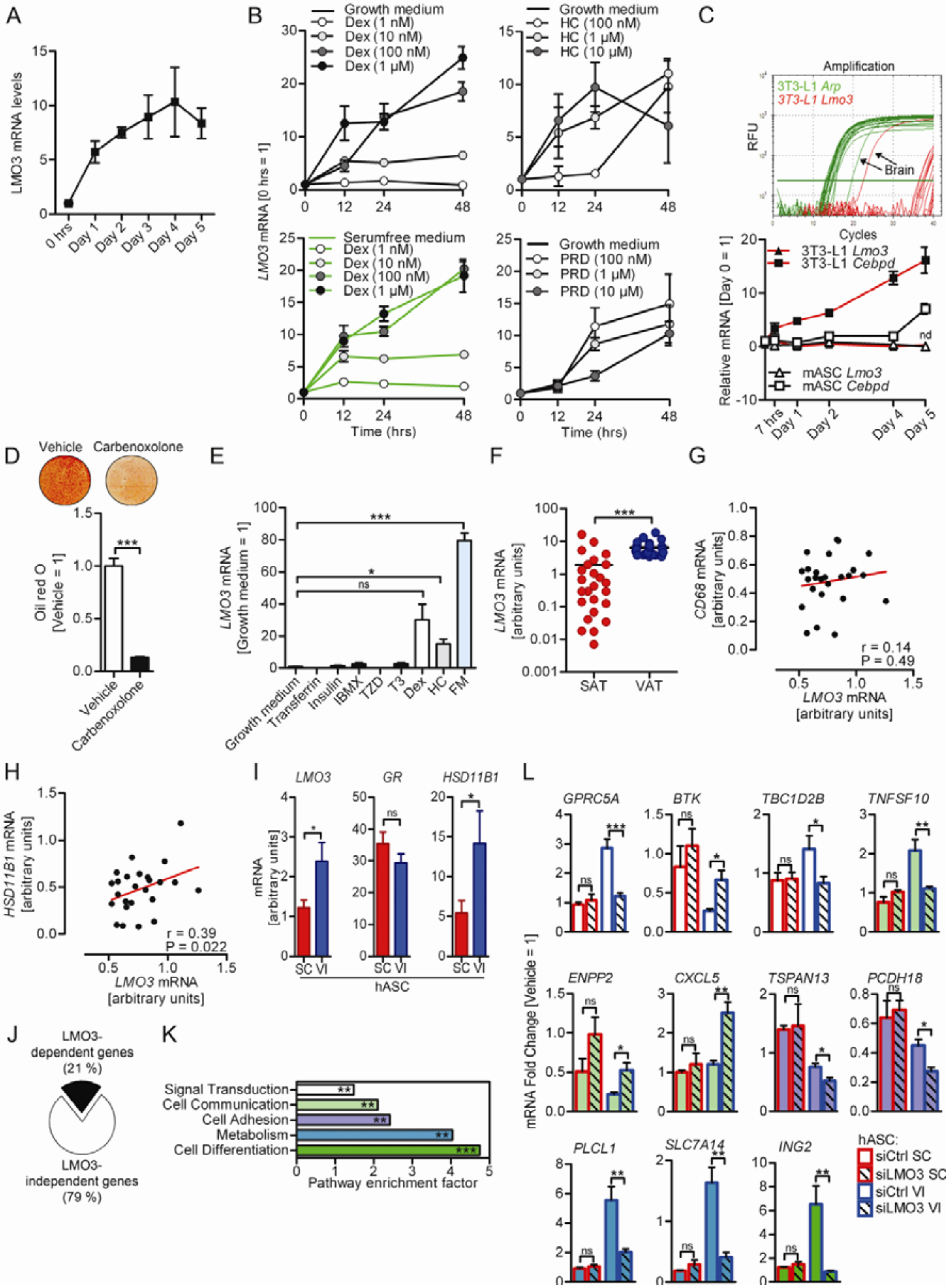

Figure S1. GC Induced LMO3 Expression in Human Adipose Depots, SGBS- and 3T3-L1 Cells, Related to Figure 1

All error bars represent means ± SEM. P values; ns=not significant; *P< 0,05, **P< 0,001 and ***P< 0,0001. (A) qPCR verification of *LMO3* mRNA expression during

hASC differentiation time-course. Differentiation induced in 2 days post-confluent cells. (B) *LMO3* mRNA levels measured in hASC after GC treatment for indicated hours. Dex, PRD and HC, were added in growth medium (GM) at indicated concentrations. Dex was additionally added in serum-free medium (SFM) at indicated concentrations. (C) Upper panel: Plotted amplification curves for respective gene upon Dex (1 μM) treatment in 3T3-L1 cells. The brain RNA lysates are from C57Bl/6J lean mice and have not been stimulated with Dex. Lower panel: Relative mRNA levels upon Dex (1 μM) stimulation throughout indicated timepoints in 3T3-L1 cells and primary mouse adipose stromal cells (mASC). nd = non detectable for day 5 3T3-L1 and mASC. (D) Human ASC treated with vehicle or carbenoxolone (100 μM) in the full mix throughout the entire differentiation. Cells were fixed and oil red O (ORO) staining was performed on day 10. Representative wells upper panel and quantification lower panel. (E) Individual components of the adipogenic cocktail were added to confluent Simpson-Golabi-Behmel Syndrome (SGBS) cells. Cells were harvested 24 hrs later for RT-PCR analysis. Full Mix (FM) refers to the differentiation cocktail. (n=3). Isobutyl-methylxanthine (IBMX); troglitazone (TZD); triiodothyronine (T3); RU486 (1 μM). All components were added in growth medium with concentrations listed under experimental procedures. (F) *LMO3* mRNA levels from 25 obese females from the matched SAT and VAT biopsies shown in figure 1L, with low *CD68* mRNA expression (G) Correlation plot shows lack of significant correlation between *LMO3* and *CD68* mRNA in the 25 subjects plotted in (F). (H) Correlation plot between *LMO3* and *HSD11B1* mRNA is apparent in the 25 subjects with low *CD68* mRNA expression seen in (F). (I) Basal mRNA expression of indicated genes in matched SC and VI matched hASC. n=6. (J) Pie chart depicting LMO3-dependent gene expression in VI preadipocytes by microarray analysis of matched SAT and VAT preadipocytes related to Figure 1O. Approximately 21 % (181 genes), were differentially regulated upon LMO3-siRNA treatment referred to as LMO3-dependent genes. (K) Pathway enrichment analysis on LMO3-dependent genes in VAT (181 in total) with DAVID and EASE. Enrichment is shown as a Z-score. (L) RT-PCR analysis of LMO3-dependent genes in VAT. Colour coding matches the gene pathway enrichment graph shown in (K).

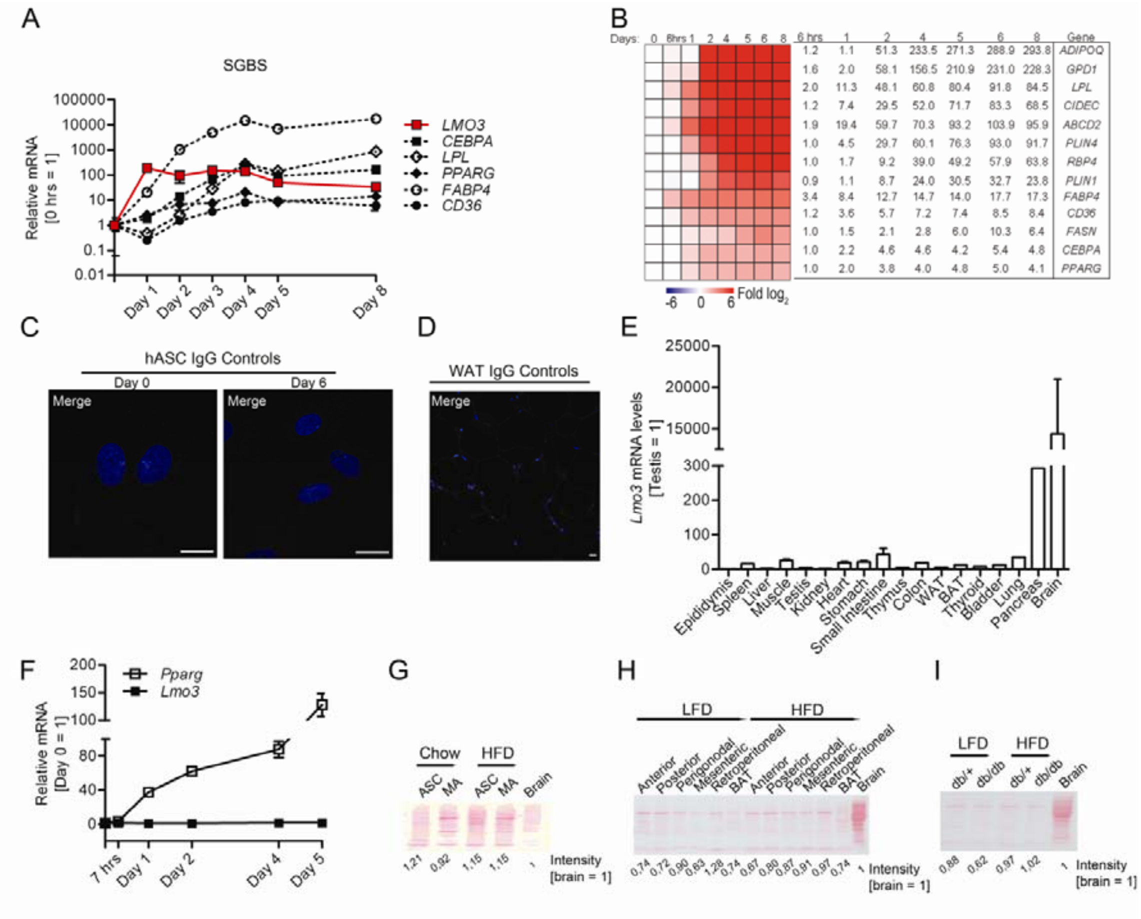

Figure S2. LMO3 Expression during Adipogenesis Is Human Specific, Related to Figure 2

All error bars represent means ± SEM. P values; ns=not significant; *P< 0,05, **P< 0,001 and ***P< 0,0001. (A) 2 day post-confluent SGBS cells were induced to differentiate into adipocytes. Total RNA was isolated at the indicated times post induction. mRNA levels of respective gene were measured by RT-PCR and normalized to the amounts of *RPLP0*. Bar graphs indicate mean fold change ± SEM of three independent experiments. (B) Verification of successful hASC differentiation, based on proadipogenic marker mRNA expressions analyzed by DNA Microarray expression profiling. Shades of red and blue indicate distinct degrees of gene activation. Fold-change activation from day 0 of differentiation is shown to the right of the heatmaps. (C) to (D) Scale bars = 20 µm. (C) Specificity of staining (seen in Figure 2H) is verified by negative isotype controls for LMO3 and perilipin in hASC on day 0 and 6. (D) Specificity of staining (seen in Figure 2I) is verified by negative isotype controls for LMO3, CD68 and perilipin in human WAT. (E) *Lmo3* mouse tissue distribution pattern evaluated by a commercially available pool of murine multiple tissue RNA library by RT-PCR analysis and normalized to the amounts of *Rplp0* in mouse. Each tissue is a pool of at least 3 donors. (F) Differentiation timecourse of 3T3-L1 cells indicating *Lmo3* and *Pparg* mRNA expression. (G) to (I) Ponceau staining as loading controls for blots shown in Figures 2K to N. Total lane intensity was quantified to brain lane.

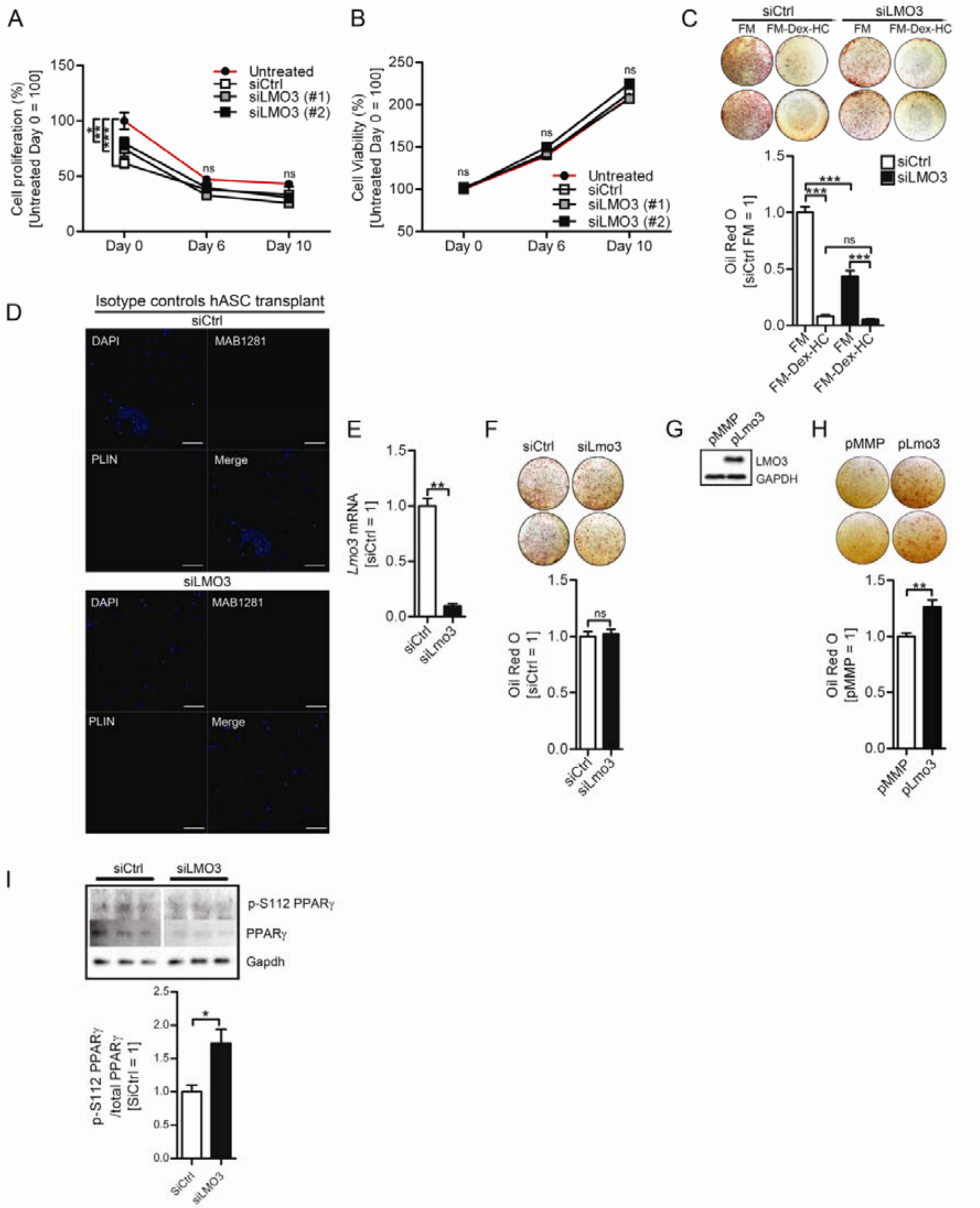

Figure S3. Impact of Silencing and Overexpressing LMO3 in hASC and Murine 3T3-L1 Cells and Phosphorylation Status of S112 PPARγ in Differentiated hASC, Related to Figures 3 and 4

All error bars represent means ± SEM. P values; ns=not significant; *P< 0,05, **P< 0,001 and ***P< 0,0001. (A) BrdU incorporation in hASC (cell proliferation) was initiated 48 hrs post transfection. Untreated cells are non-transfected. Differentiation timepoints chosen to match Figure 3. (B) MTT assay evaluating cell viability of untransfected (untreated) and siOligo transfected hASC. Assay was initiated 48 hrs post transfection. Differentiation timepoints chosen to match Figure 3. (C) Human ASC were subjected to siOligos; siCtrl or siLMO3 then post 48 hrs the FM with or without GCs (Dex and HC, both at 1 μM), throughout the entire differentiation. Cells

were fixed and lipid droplets stained with Oil red O on day 18 (representative wells upper panel, magnification 10X) and quantified (lower panel). (D) Specificity of staining (seen in Figure 3F) is verified by negative isotype controls for human nuclei specific marker MAB1281 and perilipin in both siCtrl- and siLMO3 injected SCID mice. Scale bar = 20 μM. (E) RT-PCR evaluating *Lmo3* mRNA levels. siCtrl or siLmo3 murine 3T3-L1 cells were harvested on day 2 of differentiation (initiated 48 hrs post transfection). (F) Oil red O staining on siCtrl or siLmo3 transfected 3T3-L1 cells. Differentiation was initiated 48 hrs post transfection and cells fixed on day 7 (representative wells upper panel, magnification 10X) and quantified (lower panel). (G) Protein analysis of empty pMMP vector or retrovirus expressing LMO3 transduced 3T3-L1 preadipocytes. (H) Oil red O staining on pMMP or pLmo3 expressing 3T3-L1 cells. Differentiation was initiated 48 hrs post transfection and cells fixed on day 10 (representative wells upper panel, magnification 10X) and quantified (lower panel). (I) Transfected and day 6 differentiated hASCs were serum starved overnight then replenished for 1 hr with 40 % FBS. Top panels are representative blots from at least three donors. Bottom panels: Densitometric evaluation.

Table S2. Anthropometric and Metabolic Characteristics of Human Study Subjects, Related to Figure 1

Trait	Non-obese[A]	Obese[A]	Individuals from obese cohort $CD68_{LOW}$[A,B]
N	7	55	25
Male/Female (n)	0/7	15/40	0/25
Age (years)	46.4 (33-57)	38.3 (17-59)	43.4 (30-56)
BMI[C] (kg/m^2)	25.2 (20.3-28.1)	43.2 (33.0-60.5)	42.2 (33.5-60.5)
Glucose, fasting (mmol/L)	5.0 (4.3-5.7)	5.2 (4.1-6.1)	5.1 (4.1-5.9)
Insulin, fasting (pmol/L)	47.5 (15.3-84.7)	128.1 (24.3-403.5)	98.4 (24.3-243.1)
CD68 mRNA (AU[C])	3.7 (1.89-6.25)	4.1 (1.28-9.89)	3.3 (1.28-5.98)

[A]Data are numbers of observations or means (range).

[B]$CD68_{LOW}$, obese female study subjects displaying *CD68* mRNA values within the minimum to maximum range of healthy, non-obese females.

[C]Abbreviations: BMI, body mass index; AU, arbitrary unit.

Table S3. Correlation of *LMO3* and *HSD11B1* mRNA Expression Levels in Fat Depots from Obese and Obese, $CD68_{LOW}$ Study subjects, Related to Figure 1

Obese Study Subjects (n=55)				
SAT[A]		VAT[A]		
Adjusted Beta/R[B]	*P*[B]	Adjusted Beta/R[B]	*P*[B]	Adjusted for
-0.110	0.426	0.268	0.048	-
-0.150	0.285	0.314	0.025	sex, age
-0.150	0.290	0.312	0.026	sex, age, BMI[A]
**Obese, C*D68*$_{LOW}$[C] Study Subjects (n=25)**				
SAT[A]		VAT[A]		
Adjusted Beta/R[B]	*P*[B]	Adjusted Beta/R[B]	*P*[B]	Adjusted for[D]
0.178	0.292	0.364	0.025	-
0.152	0.416	0.395	0.022	Age
0.151	0.435	0.372	0.028	age, BMI[A]
0.133	0.538	0.427	0.044	age, CD68
0.131	0.553	0.397	0.049	age, BMI[A], CD68

[A]Abbreviations: SAT, subcutaneous adipose tissue; VAT, visceral adipose tissue; BMI, body mass index.

[B]Standardized beta-coefficients and *P* values obtained from regression analysis. Linear regression adjusted for indicated variables. All variables normally distributed before or after $\log_{10}$-transformation.

[C]$CD68_{LOW}$: obese female study subjects displaying *CD68* mRNA values within the minimum to maximum range of healthy, non-obese females.

[D]No sex adjustment as all study subjects are female.

Table S5. Characteristics of the Non-obese, Obese Low HOMA-IR and Obese High HOMA-IR Study Participants, Related to Figure 1

Trait	Non-Obese	Obese low HOMA-IR	Obese high HOMA-IR	P*	P†	P‡
Male/female (n)	0/7	4/18	8/18			
Age (years)	46.2 (3.5)	39.6 (2.2)	37.1 (2.3)	n.s.	n.s.	n.s.
BMI (kg/m^2)	25.2 (1.2)	41.8 (1.4)	44.4 (0.9)	n.d.	n.d.	n.s.
HOMA-IR	1.6 (0.4)	1.5 (0.1)	7.2 (0.5)	n.d.	n.s.	n.d.
Waist circumference (cm)	88.5 (4.5)	119.5 (3.1)	132.0 (3.0)	n.d.	n.d.	0.033
Insulin (pmol/L)	47.5 (10.0)	47.0 (2.2)	210.6 (14.0)	n.d.	n.s.	0.000
Glucose (mmol/L)	5.0 (0.2)	4.8 (0.1)	5.4 (0.1)	n.d.	n.s.	0.000
*LMO3*mRNA, VAT (AU)	8.72 (2.90)	8.32 (1.32)	8.05 (0.80)	n.s.	n.s.	n.s.
LMO3 mRNA, SAT (AU)	0.49 (0.13)	2.06 (0.82)	0.77 (0.18)	n.s.	n.s.	n.s.
HSD11B1 mRNA, VAT AU)	0.35 (0.07)	0.39 (0.06)	0.75 (0.06)	0.000	n.s.	0.000
HSD11B1 mRNA, SAT AU)	0.46 (0.13)	0.73 (0.12)	0.63 (0.08)	0.000	n.s.	0.000

Data are numbers of observations or unadjusted and untransformed means (SEM). Two-sided P values obtained from ANOVA (Scheffé-Test for subgroup comparisons adjusted for age and sex). Heteroskedasticity tested by the Breusch-Pagan/Cook-Weisberg test, normal distribution of quantitative traits tested by the Shapiro-Francia normality test. Non-normal distributed parameters transformed ($\log_{10}$ transformation for HOMA-IR, insulin, *LMO3* mRNA and *HSD11B1* mRNA); *comparison among all groups; †non-obese controls vs. obese low HOMA-IR; ‡obese low HOMA-IR vs. obese high HOMA-IR; participants (total) n = 55 (7/22/26; non-obese/obese low HOMA-IR/obese high HOMA-IR); low HOMA-IR, HOMA-IR <=2.0; high HOMA-IR, HOMA-IR >=5.0; n.s., not significant; n.d., not determined; VAT., Visceral Adipose Tissue; SAT, Subcutaneous Adipose Tissue, AU, arbitrary units.

Table S6. Correlations between Visceral Adipose *LMO3* mRNA and Anthropometric and Metabolic Characteristics of Non-obese and Obese Study Subjects, Related to Figure 1

Trait	Beta/R*	P*	Beta/R†	P†
BMI (kg/m^2)	-0.157	n.s.	-0.032	n.s.
HOMA-IR	0.092	n.s.	0.179	n.s.
Waist circumference (cm)	-0.140	n.s.		n.d.
Insulin (pmol/L)	0.092	n.s.	0.180	n.s.
Glucose (mmol/L)	0.048	n.s.	0.094	n.s.
HSD11B1 mRNA, VAT. (AU)	0.284	0.031	0.359	0.010

Standardized beta-coefficients (Beta/R) and two-sided P values obtained from regression analysis. Normal distribution of traits tested by the Shapiro-Francia normality test. Non-normal distributed parameters transformed ($\log_{10}$ transformation for HOMA-IR, insulin, *LMO3* mRNA and *HSD11B1* mRNA).

*adjusted for age and sex; †adjusted for age, sex and waist circumference; participants (total) n = 62 (7 non-obese/55 obese); n = 59 (4/55) for waist circumference and waist circumference adjusted;

Abbreviations: n.s., not significant; n.d., not determined; VAT., Visceral Adipose Tissue., visceral; AU, arbitrary units.

SUPPLEMENTAL EXPERIMENTAL PROCEDURES

Isolation of Preadipocytes Extended Procedure

Minced adipose tissue was washed in DMEM (PAA) thereafter digested with 1 mg/ml collagenase type II (Worthington) and 50 units/ml deoxyribonuclease I (Sigma) in DMEM, supplemented with 50 µg/ml gentamicin (Invitrogen) for 1 hr at 37°C with constant shaking. Cells were filtered through 100 µm nylon filters and centrifuged. Floating cells were taken as mature adipocytes. Erythrocytes were lysed in hypotonic buffer (Qiagen), thereafter centrifuged and cell pellet resuspended in growth medium: DMEM/Ham's F12 (PAA), 50 µg/ml gentamicin and 10 % fetal bovine serum (PAA). After filtration using a 20 µm nylon mesh, cells were incubated at 37°C with 5% CO_2 for 24-48 hrs.

Mouse Studies Extended Information

C57BL/6J mice were fed chow, LFD or HFD for 10 weeks; db/db and db/+ mice LFD or HFD for 20 weeks (LFD D12450B, HFD D12492; Research Diets Inc.) (diets administered ad libitum).Mice were sacrificed and depots excised and snap frozen in liquid N_2 prior to further processing. Weights: C57BL/6J LFD: 33.3 ±1.6 g, chow: 35.9 ±5.0 g, HFD: 47.9 ±3.6 g; db/db LFD 46 ±0.7 g, HFD 54.7 ±1 g, and db/+ LFD: 29.3 ±0.5 g, HFD 31.1 ±0.8 g.

RT-PCR

RT-PCR was performed as described in main article under experimental procedures. Primer sequences are listed in Table S9. All RT-PCR data are normalized to amounts of acidic ribosomal phosphoprotein P0 (*RPLP0*), unless otherwise stated. An in-house made mouse tissue library was used to analyze the tissue distribution of mouse *Lmo3* expression.

Western Blot Analyses Extended Information

PVDF membranes (GE healthcare) were incubated with following antibodies: LMO3 (Abnova), LMO3 (Santa Cruz), TopoIIβ (BD Biosciences), GAPDH (Santa Cruz), V5 antibody (Invitrogen), p-p44/42 MAPK (pERK1/2) (Cell Signaling), p44/42 MAPK (ERK1/2) (Cell Signaling); HSP70 (Cell Signaling), PPARγ (Santa Cruz), p-S112-PPARγ (Abcam), CEBPβ (Santa Cruz), Perilipin, (Cell Signaling), MAC2 (Cedarlane) and CEBPα (Santa Cruz). Hrp-conjugated IgG secondary antibodies were used (Cell Signaling) and blots were developed with ECL Plus Western Blotting Detection System (GE Healthcare). TotalLabQuant software (TotalLab Limited) was used for densitometric quantification.

Gene Expression Profiling Extended Information

GC target genes were identified with a time-course in hASC cells treated with Dex . Genes with signal intensities below 100 were eliminated. Genes exhibiting ±3-fold change relative to control treatment (in at least three out of five comparisons) were selected for further analysis. This approach identified 121 up- and 90 downregulated genes in response to Dex treatment in hASC.

LMO3 target genes following GC stimulation were identified with duplicate well RNA pooled for GeneChip analysis (1 chip per time point) following a published 2-step protocol (Asada et al., 2011): First, genes responsive to the adipogenic cocktail in siCtrl-treated (control) hASC with mean ±1.5-fold expression day 6 vs. 0 of differentiation, yielded a set of 1892 adipogenesis-regulated genes. Next, from these 1892 genes we identified LMO3-dependent genes by comparing fold-changes (day 6 vs.0) of siCtrl with LMO3 knockdown (siLMO3 #1 or siLMO3 #2) hASC. Genes were

classified as LMO3-dependent if the fold-change was ±1.5–fold different between siCtrl- or siLMO3 transfected hASC. DAVID and EASE algorithm was used to identify specific biological pathways. The results
were visualized using GenespringGX (Agilent, Santa Clara, CA).

Human and Murine Primers and Silencing Oligonucleotide-Sequences.
siOligo sense sequences: *LMO3 #1,* 5‘-GGUAUCUUCUAAAGGCACUGGACAA-3‘;*LMO3 #2,* 5‘-CCCUGUACACUAAAGCUAAUCUUAU-3‘;*GR,* 5’-GCAUGUACGACCAAUGUAA-3’; *PPARG,* 5’-GGGCGAUCUUGACAGGAAATT-3’; *Lmo3,* 5’-GGAGACAAAUUUUUCUUAATT-’3. Genes forward- followed by reverse primer sequences: *ADIPOQ,* GCTGGGAGCTGTTCTACTGC, CGATGTCTCCCTTAGGACCA; *BTK,* GTCCTGGCATCTCAATGCATC, AATCACTGCGGCCATAGCTTC; *CD36,* GGGAAAGTCACTGCGACATGAT, ACGTCGGATTCAAATACAGCATAGA; *CD68,* GCTACATGGCGGTGGAGTACAA, ATGATGAGAGGCAGCAAGATGG; *CEBPA,* CTTGTGCCTTGGAAATGCAA, GCTGTAGCCTCGGGAAGGA; *CXCL5,* GACCACGCAAGGAGTTCATCC, AGGCTACCACTTCCACCTTGG; *ENPP2,* ATCTTCTCCGTGGATGGCTTC, GAGAGTGTGTGCCACAAGACC; *FABP4,* AAGGCGTCACTTCCACGAGAG, AATGCGAACTTCAGTCCAGGTC; *FMN1,* CCAATTCTAAGCCAGCGGATG, CAGCTTGAAGTCTGCCAGGAG; *GPRC5A,* CAAGGTGCAGGACTCCAACAG, ATGATGAAGGCGAAGGTGAGG; *HSD11B1,* TCATTCTCAACCACATCACCAACACT, CCAGCCAGAGAGGAGACGACAAC; *ING2,* CAGCAGCAGCAGCAACTGTAC, TCCACGCACTCAAGGTAGTCC; *LMO3,* AAGGTTGTGCTGGCTGCAAC, GGCACACTTCAGGCAGTCTTC; *LPL,* TGGAGGTACTTTTCAGCCAGGAT, TCGTGGGAGCACTCACTAGCT; *PLCL1,* CTGTCATCTCGGCTCATCACC, TCTGCACATCTGGAATTGCAC; *PLIN1,* GACAACGTGGTGGACACAGT, CTGGTGGGTTGTCGATGTC; *PPARG1,* AAGGCCATTTTCTCAAACGA, AGGAGTGGGAGTGGTCTTCC; *PPARG2,* CCATGCTGTTATGGGTGAAA, TCAAAGGAGTGGGAGTGGTC; *PCDH18,* CTCCAGTGTGCAGCCTTCTTC, CCATCATTGAGGTGGTTGAGC; *PSPH,* TGGTGCCACAGATATGGAAGC, GGCGTTATCCTTGACTTGTTGC; *RPLP0,* GTCATCCAGCAGGTGTTCGAC, CTCCAGGAAGCGAGAATGCAG; *SLC7A14,* ATCGCAGGCCTCTTCTTCATC, GAAGCATGTTGCTGCTCCTTG; *TBC1D2B,* AATGCAACTGCAGGTCCAGAG, CTGGAGCAGTCGAACAAGCTC; *TNFSF10,* GCTCCTGCAGTCTCTCTGTGTG, GTCCTGCATCTGCTTCAGCTC; *TSPAN13,* CTTCGCGTGTTCCAAGAACTG, CCACGCAGCAATTCCAATTAG; *VNN1,* CTGGTGGCACGCTACCATAAG, TCACAATCTCAGGCTCCTTGG; *Lmo3,* GCATGAGGACTGCCTGAAGTG, GCCTTCGTGTACAAGGTGGAG; *Pparg,* GCATGGTGCCTTCGCTGA, TGGCATCTCTGTGTCAACCATG; *Cebpd,* ATCGACTTCAGCGCCTACAT, GCTTTGTGGTTGCTGTTGAA; *Rplp0,* GCCAATAAGGTGCCAGCTGCTG, GAAGGAGGTCTTCTCGGGTCCTAG

Luciferase Assays Extended Information
For LMO3-promoter activity assays 293FT cells were co-transfected in 48-wells with either 50 ng of a LMO3 luciferase reporter plasmid (Switchgear Genomics) or a 5 point mutated LMO3 luciferase reporter plasmid within the human GRE1 site along with 250 ng of a GR expression plasmid (Addgene) or control DNA (pcDNA3.1, Invitrogen; pcDNA was added for a consistent amount of DNA and set to 1 for comparison). 10 ng of pCMV-β-gal expression plasmid was used as control for transfection efficiency. Point mutations to generate pLMO3-Luc-Hs.->Mm was performed by site-directed mutagenesis with the QuikChange Multi Site Directed Mutagenesis kit (Stratagene). The mutations within the human GRE1 sequence were

generated using a mutagenic primer: 5'-CCCATCCGCACAGCTCCCTgcTtgTCcACCCCACCTCTCTGCTAC-3' (upper strand sequence between −951 and −906 bp; the lowercase letters indicate nucleotide substitutions). DNA sequencing verified the subcloned fragment. For PPARG-reporter assays 250 ng PPARG-responsive reporter plasmid p(AOx)3-TKSL (provided by Christopher K Glass, University of California, San Diego, CA, USA), 250 ng PPARG2 expression plasmid (pPPARG) or 250 ng PPARG-S112A expression plasmid (pPPARG-S112A), 50, 100 or 250 ng pLMO3-V5 expression plasmid (Origene), or 250 ng pMCL-HA-MAPKK1-11A55B [S218E/S222D] expression plasmid (Addgene) and 10 ng Renilla luciferase control plasmid (ph-RL; Promega) were transfected into cells using Lipofectamine 2000 (Invitrogen).
ELK-1 activity was measured using the luciferase reporter gene assay by transfecting 293FT cells with plasmids containing Gal4-UAS-luciferase (pFR-Luc) and a fusion of activation domain of ELK-1 and Gal4 DNA-binding domain (DBD) (pFA2-ELK-1). ELK-1 activation in the nucleus causes binding of Gal4-DBD to Gal4-UAS, leading to luciferase transcription. Cell were cotransfected with Renilla luciferase control plasmid (ph-RL; Promega). 293FT cells cultured on 24-well plates (plated at 5 × 105/well density) were cotransfected with pFR-Luc (0.3 µg/well), pFA2-ELK-1 (0.3 µg/well), phRL (0.02 µg/well), pLMO3 or control DNA (pcDNA3.1) using Lipofectamine 2000. 24 hr after transfection, cells were treated with EGF (Sigma,100 ng/ml) for 20 h. Luciferase activities were measured using the Dual-Luciferase Reporter Assay System (Promega) or the LightSwitch Luciferase Assay (Switchgear Genomics) and a multimode microplate reader (Synergy 2, BioTek Instruments). Luminescence values were normalized to Renilla luminescence levels or ß-Galactosidase activity to the ß-Galactosidase Enzyme Assay System (Promega), respectively.

Oil Red O Staining
Cells were fixed in 10% formalin, thereafter stained with Oil Red-O (Sigma) working solution (composed of 4 parts water and 6 parts 0.6% Oil red O dye in isopropanol). Quantification was performed by eluting stained cells and measuring optical density using a multimode microplate reader (Synergy 2, BioTek Instruments).

Confocal immunofluorescence Microscopy Extended Information
HASC were seeded in matrigel (BD Biosciences) pre-coated chamber slides and differentiated then fixed with cold methanol. Blocking: Antibody diluent with background reducing components (Dako) and human IgG (Jackson ImmunoResearch Laboratories). Antibodies: LMO3 (Abnova) and Perilipin (Cell Signaling) or respective IgG control (Sigma-Aldrich). Secondary fluorescent labeled antibodies were used for visualization (both Invitrogen). Human VAT was fixed in 10% formalin (Sigma) then paraffin embedded. Paraffin sections were deparaffinized followed by antigen retrieval by boiling in citrate buffer (Dako). Sections were blocked with 3% bovine serum albumin (BSA) then incubated with: LMO3 (Santa Cruz), Perilipin (Cell Signaling), CD68 (Dako) or respective IgG Control (all Sigma). Additional blocking step with goat serum (Invitrogen) prior to TRITC (Jackson Immunotech) was performed. Thereafter remaining secondary antibodies: Cy5 (Jackson Immunotech) and Alexa 488 (Invitrogen) followed by 4',6-diamidino-2-phenylindole (DAPI, Sigma). All antibodies were diluted in 3% BSA solution.

SCID Mouse Xenotransplant Model Extended Information.
Female, 6-week-old Crl:SHO-*Prdkc*^{scid}*Hr*^{hr} mice were obtained from Charles River (Sulzfeld, Germany) and housed as previously described (Loewe et al., 2006). A

previously published protocol was followed with minor modifications (Ahfeldt et al., 2012). In brief, siCtrl- or siLMO3 transfected hASC were differentiated for 2 days. Thereafter injected subdermally into the flank of each mouse (n=5 per group; 150,000 cells per injection). 6 weeks post injection, mice were sacrificed and transplantation tissue-site was collected, paraffin embedded, sectioned and stained. 4 sections were chosen for each mouse on the basis of when the sections transversed the tissue (light microscopy evaluation). Sections were stained for 24 hrs at 4°C with MAB1281 and with Perilipin for the last hour. Thereafter secondary antibodies and DAPI was added. In total 120 fields per view were quantified (3 fields of view per section) by counting double positive stain for MAB1281 and perilipin in relation to positive perilipin stained cells in a blinded manner.

MTT Cell Viability Assay

Untreated, siCtrl, siLMO3 (#1) and (#2) hASCs were plated into 96-well culture plates at a density of 10,000 cells per well. 2 days post amaxa differentiation was initiated and cell viability was established using MTT Cell Viability Assay Kit (Biotium) according to manufacturer’s protocol on indicated days. In brief; 10 µl MTT solution was added to 100 µl culture medium. After 4 hrs of incubation at 37°C, 200 µl dimethylsulfoxide (DMSO) was added to each well, carefully pipetting up and down to dissolve formazan. Absorbance was measured on a multimode microplate reader (Synergy 2, BioTek Instruments) at 570 nm and a reference wavelength of 630 nm.

BrdU Cell Proliferation Assay

Untreated, siCtrl, siLMO3 (#1) and (#2) hASCs were plated into 96-well culture plates at a density of 10,000 cells per well. 2 days post amaxa differentiation was initiated and cell proliferation was established using the Cell Proliferation ELISA, BrdU kit (Roche) according to manufacturer’s protocol. Plates were subsequently kept at 4°C prior to harvest of day 10 and assay was then performed. In brief, 10 µl BrdU labelling solution was added to 100 µl differentiation medium per well. After 4 hrs of incubation at 37°C, labelling medium was removed and 200 µl/well FixDenat was added to the cells for 30 minutes at RT. Solution was removed by tapping, thereafter 100 µl/well anti-BrdU-POD working solution was added for 90 minutes at RT. Wells were rinsed with washing solution 3 times, then 100 µl/well substrate solution was added for 15 minutes at RT, followed by adding 1M H_2SO_4 stop solution 25 µl/well and absorbance was measured on a multimode microplate reader (Synergy 2, BioTek Instruments) at 450 nm and a reference wavelength of 690 nm.

Retrovirus Preparation and Infection

Retrovirus preparation and infection were performed as described (Bilban et al., 2008). Human LMO3 cDNA in pDEST-51 vector (Origene) was subcloned into the retroviral vector pMMP (kindly provided by Dr. Klaus Schmetterer, Department of Laboratory Medicine, Medical University of Vienna, Vienna, Austria) with 5´ HindIII and 3´ NotI (Invitrogen, Carlsbad, CA) restriction enzymes. Confirmation was verified by restriction site analysis and sequencing. Briefly, pMMP empty vector or pMMP vector containing LMO3 cDNA, along with vectors containing reverse transcriptase (gag-pol) and VSV-G-expressing plasmids, was transfected into 293FT packaging cells with Lipofectamine 2000 (Invitrogen). Viral supernatant was collected 48 hrs after transfection, filtered through 0.45 µm filters, and added to target cells for 12 hrs along with 8 g/ml Polybrene. GFP+ cells were sorted on a FACSAria (Becton Dickinson, San Jose, CA, USA) to make stable lines and were maintained in media containing appropriate antibiotics.

SUPPLEMENTAL REFERENCES

Ahfeldt,T., Schinzel,R.T., Lee,Y.K., Hendrickson,D., Kaplan,A., Lum,D.H., Camahort,R., Xia,F., Shay,J., Rhee,E.P., Clish,C.B., Deo,R.C., Shen,T., Lau,F.H., Cowley,A., Mowrer,G., Al-Siddiqi,H., Nahrendorf,M., Musunuru,K., Gerszten,R.E., Rinn,J.L., and Cowan,C.A. (2012). Programming human pluripotent stem cells into white and brown adipocytes. Nat. Cell Biol. *14*, 209-219.

Asada,M., Rauch,A., Shimizu,H., Maruyama,H., Miyaki,S., Shibamori,M., Kawasome,H., Ishiyama,H., Tuckermann,J., and Asahara,H. (2011). DNA binding-dependent glucocorticoid receptor activity promotes adipogenesis via Kruppel-like factor 15 gene expression. Lab Invest *91*, 203-215.

Loewe,R., Valero,T., Kremling,S., Pratscher,B., Kunstfeld,R., Pehamberger,H., and Petzelbauer,P. (2006). Dimethylfumarate impairs melanoma growth and metastasis. Cancer Res. *66*, 11888-11896.
